# On Some of the Most Important Remedies Used in Brain Disease

**Published:** 1873-02

**Authors:** William Hammond

**Affiliations:** Professor of Diseases of the Mind and Nervous System in the Bellevue Hospital Medical College, New York


### ON SOME OF THE MOST IMPORTANT REMEDIES USED IN BRAIN DISEASE.

BY WILLIAM HAMMOND, M.D.,

*Professor of Diseases of the Mind and Nervous System in the Bellevue Hospital Medical College, New York.*

("A Treatise on Diseases of the Nervous System," 1871. 8vo. pp. 754.)

The following observations on some of the most important remedies used in brain disease will be found deserving of attention:

"Bromide of potassum can almost always be used with advantage to diminish the amount of blood in the brain, and to allay any excitement of the nervous system that may be present in the sthenic form of insomnia. That the first named of these effects follows its use, I have recently ascertained by experiments upon living animals, the details of which will be given hereafter. Suffice it now to say, that I have administered it to dogs whose brains have been exposed to view by trephining the skull, and that I have invariably found it to lessen the quantity of blood circulating in the cranium, and to produce a shrinking of the brain from this cause. Moreover, we have only to observe its effects upon the subject, to be convinced that this is one of the most important results in employment. The flushed face, the throbbing of the carotids and temporals, suffusion of the eyes, the feeling of fullness in the head, all disappear as if by magic under its use. It may be given in doses of from ten to thirty grains, the latter quantity being seldom required, but may be taken with perfect safety in severe cases.

"Since then, experiments with the cephalo-hæmometer and ophthalmoscope have abundantly confirmed these views, and more extensive experience in the treatment of cerebral congestion has placed the matter beyond the possibility of a doubt. Other observers have also confirmed the opinions here expressed.

"The prescription which I usually employ consists of bromide of potassium, ℥ j.; water, ℥ iv.; of this, a teaspoonful is taken three times a day in a little water. Occasionally, the bromide is increased to ℥ iss., and sometimes a saturated solution—which contains gr. xxx. to ʒ j.—is used. I continue the medicine till drowsiness, a slight feeling of weakness in the legs, and contraction of the blood vessels of the retina—detected by the ophthalmoscope—are produced. The more prominent head symptoms generally disappear in four or five days, and the results above mentioned ensue in about ten days.

" Latterly, I have used the bromide of sodium in corresponding doses, instead of the bromide of potassium. It is more pleasant to the taste, and does not cause so much constitutional disturbance as sometimes follows the administration of the bromide of potassium in large doses.

"In conjunction with one or other of the bromides mentioned, I very generally employ the oxide of zinc, which experience has taught me is a powerful agent in relieving cerebral congestion, and giving tone to the nervous system. It should be given in doses of gr. ij., three times a day, either in the form of a pill or a powder, and, to avoid any nausea, should be taken after meals. At the end of about ten days it will generally be found that all symptoms of congestion—subjective and objective—have disappeared, leaving a little debility and mental depression. It then becomes expedient to give tonics and restoratives, and those which have a special action on the nervous system are to be preferred. Among them strychnia, phosphorous and cod-liver oil stand first.

"Strychnia may be advantageously administered in conjunction with iron and quinine, dissolved in dilute phosphoric acid, as in the following formula: Strychniæ sul., gr. j.; ferri pyrophosphatis, quiniæ sul., aa. ℨj.; acid. phos. dil., zingiberis syrupi, aa. ℥ ij. M. ft. mist. Dose, a teaspoonful three times a day in a little water. I prefer this extemporaneous prescription to any of the syrups or elixirs with like ingredients. If for any reason the iron and quinine are not indicated, the strychnia can be given alone with the dilute phosphoric acid.

"Phosphorus almost always acts well in such cases as those under consideration. It may be given in the form of the phosphorated oil, as in the following formula: ℞.—Olei phosphorat, ℥ ss.; mucil. acaciæ, ℥j.; olei bergami, gtt. xl. M. ft. emulsio. Dose, gtt. xv. three times a day. A very elegant preparation of phosphorus is the phosphide of zinc. . . . . My experience with this medicine has been very extensive. I have never known it to produce the least unpleasant effect, and have rarely been disappointed in obtaining the full results to be expected from phosphorus in corresponding doses. I am, therefore, not in accord with Dr. M. Clymer on this point.

"The chemical formula of phosphide of zinz is PZn3, and consequently a grain represents a little more than one-seventh of a grain of phosphorus. The proper dose, therefore, is about the tenth of a grain. I usually prescribe it in cerebral congestion, according to the following prescription: ℞.—Zinci phosphidi, gr. iij.; rosar. conserv. q. s. M. ft. in pil. no. xxx. Dose, one three times a day. Instead of the conserve of roses, gr. x. of the extract of nux vomica may be substituted if strychnia is not being administered in some other form."—*Half-Yearly Abstract.*

### OZÆNA.

Dr. Prosser James, in a paper read before the London Medical Society, after alluding to the intractability of this affection, and the almost utter hopelessness with which it is too often regarded, divides it for consideration into catarrhal, syphilitic and accidental varieties, and says of treatment:

In every case, local measures should be resorted to, and, in the majority, constitutional remedies are indispensable. The first point is, to clear away the discharges. Until this is accomplished, the diagnosis itself cannot be made. "What," says the author, "can the most skillful use of the rhinoscope show on a surface covered with discharge?" The use of the nasal douche was said to be then the first measure. This will, probably, have to be continued assiduously. At first, common salt, chlorate of potash, carbonate of soda, or other alkali, should be used in the proportion of a teaspoonful to a pint or a quart of tepid water. Other substances had also been used by the author, and amongst them chloride of alumnium. This gave fair results, but the best remedy was a permanganate. The comfort this gives to the patient is remarkable, and, under its persistent use, the membrane assumes a healthy appearance. It at once removes the fœtor in many cases, and this is all in all to the patient. A weak solution may be employed at first, gradually increasing it until it produces a little smarting, for it should not be forgotten that this substance is a powerful caustic, one of the best and safest we can employ. Ulcerations and erosions may be touched with a strong solution or with a paste, and the whole membrane thoroughly and frequently washed with a weak solution by means of the nasal douche, the atomizer, and camelhair brushes. Mercurial lotions are used by some, but are not so effectual as permanganates, and the risk of absorption, after the recent case of a far more justifiable resort to the mineral, cannot be overlooked. Various powders by insfflation are sometimes effectual, though this mode of medication has serious drawbacks. Inhalation of vapors, especially that of iodine, is often of great value. Of course, small abscesses are to be opened, pieces of exfoliated bone removed, and other ordinary indications carried out. With regard to constitutional treatment, scrofula has already been mentioned. Anæmia and cachexia may be present, and, if so, is of the greatest importance. Then, as to syphilis. It must always be treated through the system. Some would use mercury for syphilitic ozæna, but the author does not employ it. Sir B. Brodie thought it hastened the separation of dead bone. Mr. Henry Lee had mentioned to the author some cases that were benefited by calomel baths. The author would rather iodize than salivate. He gave iodide of potassium in large doses. The dose is to be measured by its effect on the disease, and the ability of the patient to bear it.

Although iodide of potassium is the most common form of administering iodine, one object of this paper was to bring before the Society the value of other preparations of the metalloid. The author had obtained important results in syphilitic diseases of the throat, nose and mouth from other iodine salts. It is clearly not the potash that cures syphilis, and, feeling this, he gave full trial to iodide of sodium. Soda is a constituent of the frame, and is always more assimilable than potash. The sodic salt is more pleasant to the taste. Weight for weight, it contains more iodine than the potassic salt. It can frequently be taken by patients who cannot tolerate the more commonly used salt. When abroad, the author learned that his experience was corroborated in the Vienna hospitals.

Iodide of calcium was also pronounced an excellent preparation. It is easily borne by the system, and much more agreeable to take. It may, in fact, be used as a substitute for table salt. It is really desirable that the profession should recognize that all the salts of iodine are not so unpalatable as the one in common use. A specimen of iodide of calcium was exhibited, which had been prepared for the author by his accomplished friend, Dr. Tichborne, of the Dublin Apothecaries' Hall. It was, when formed, a beautiful crystalline mass, but had been broken up. The iodides of sodium and calcium were introduced to the Society because, though often used abroad, they had not been much employed in this country, while the iodide of ammonium, since being introduced by Dr. Richardson, was frequently used. Idoform was also mentioned by the author, but its therapeutic properties seemed to require further investigation.—*Doctor.*

---

MORPHIA AS A PARTURIENT.—Dr. Urmston, of Illinois, reports to the Cincinnati *Clinic* the case of a female to whom he administered morphia for the purpose of suspending a painful and ineffectual labor. To his amazement, the result was rapid dilatation of the os and speedy delivery. He attributes the phenomenon to the peculiar influence of morphia in removing a supposed rheumatism of the womb. We advise him to go on with the practice, and he will find similar results when there is no room to suspect rheumatism. In a slow and painful labor, with little disposition to relaxation of the os uteri, a large dose of morphia will almost invariably prove an active parturifacient. It relieves the pain, and while it appears to arrest labor, relaxation takes place, and the child steals a march on the accoucheur.—*Pacific Medical and Surgical Journal.*

